# The impact of chronic stress on cortical thickness in patients with depression

**DOI:** 10.3389/fpsyt.2025.1554476

**Published:** 2025-07-08

**Authors:** Na Li, Yong Li, Yueying Lu, Kun Zhang, Shuo Wang, Chaomin Wang

**Affiliations:** ^1^ Department of Psychiatry, the First Hospital of Hebei Medical University, Shijiazhuang, Hebei, China; ^2^ Department of Clinical Psychology, the First Hospital of Hebei Medical University, Shijiazhuang, Hebei, China

**Keywords:** chronic stress exposure, depression, cortical thickness, HAMD-17, stress-related brain changes

## Abstract

**Objective:**

To investigate the effect of chronic stress on cortical thickness in patients with depression.

**Methods:**

We recruited 90 participants (Jan 2023-Oct 2024): 30 treatment-naive depressed patients with chronic stress (CSD group; mean age 39.1 ± 9.8 years, baseline HAMD-17 27.4 ± 4.8), 30 treatment-naive depressed patients without chronic stress (Dep group; 38.9 ± 9.6 years, HAMD 26.1 ± 4.9), and 30 age/sex-matched healthy controls (HC; 41.2 ± 10.0 years). Chronic stress was defined using the Life Events Scale (LES > 100, based on regional norms). All patients received Fluvoxamine Maleate treatment. LES scores, HAMD-17 scores, and cortical thickness (via MRI) were assessed pre-treatment and 2 months post-treatment. Depressive relapse was monitored for 12–14 months (mean 12.9 ± 1.6 months).

**Results:**

Baseline: LES scores were CSD > Dep > HC (all P<0.05); HAMD scores were CSD≈Dep > HC (P<0.05). Compared to HC, both patient groups showed reduced baseline cortical thickness in left pars triangularis, pars opercularis, precuneus, middle temporal, and cingulate gyri, and right superior frontal, precuneus, and inferior frontal gyri (P<0.05). Crucially, the CSD group exhibited significantly greater thinning than the Dep group in these regions (P<0.05). Post-treatment (2 months): LES and HAMD scores decreased significantly in both patient groups but remained higher than HC (P<0.05). Post-treatment LES scores remained CSD > Dep (P<0.05), while HAMD scores were similar between patient groups (P>0.05). Cortical thickness increased in CSD and Dep groups but did not reach HC levels (P<0.05), and the difference between CSD and Dep thickness was no longer significant (P>0.05). The CSD group experienced significantly faster time to relapse (mean 5.4 ± 2.3 months) compared to the Dep group (8.3 ± 2.5 months; t=4.656, P<0.001).

**Conclusion:**

Chronic stress contributes to greater cortical thinning in depressed patients, particularly in specific frontal, temporal, parietal, and cingulate regions. Although treatment facilitates cortical thickness recovery alongside symptom improvement, individuals experiencing chronic stress relapse more quickly.

## 1 Introduction

Depressive disorders are characterized by persistent low mood and are among the most common mood disorders, with depression being the most prevalent type. Patients typically present with symptoms such as emotional dullness (or low mood), loss of interest, and cognitive decline, often experiencing recurrent episodes and prolonged courses of illness (1). According to a report by the World Health Organization (WHO) (2), approximately 350 million people globally suffer from depression, and the annual incidence rate of depression is approximately 4.7%. In China, the lifetime prevalence of depressive disorders is 6.8%, and the lifetime prevalence of major depressive disorder is 3.4% (3).

The exact pathophysiology of depression remains unclear. However, current theories suggest that the development of depression results from a complex interaction between genetic factors, neurocellular changes, hormonal fluctuations, (such as sex hormones), and external stressors, including stressful life events and substance abuse (4). The concept of stress was first introduced by Canadian pathophysiologist Hans Selye, who defined it as a physiological and psychological response of the body to environmental stimuli (5).

The physiological mechanisms underlying stress primarily involve two key systems: the sympathetic-adrenal-medullary (SAM) axis and the hypothalamic-pituitary-adrenal (HPA) axis. These systems affect numerous bodily functions, including executive cognitive processes, growth, reproduction, emotions such as reward and fear, sleep and wakefulness, as well as digestive, circulatory, metabolic, and immune system functions. Psychological research indicates that stress is an adaptive response of the body to external stimuli. While individuals can often gradually adapt, when chronic stress persists and damages the central nervous system, it can impair adaptive capacity and potentially cause various physiological and psychological problems (6–8).

Previous studies have highlighted the significant impact of chronic stress on neuronal morphology and function, particularly in brain areas such as the amygdala and hippocampus, which are involved in emotional processing, recognition, and regulation. This suggests that chronic stress may be an important potential trigger for the development of depression (9). While depression was once considered purely a functional disorder, advancements in neuroimaging have demonstrated structural changes in the brains of depressed patients (9).

Despite the growing body of research on the effects of depression on brain structure, it is important to note that depression may result from abnormalities in brain network structures. However, most imaging studies have focused on specific brain areas or volume changes. Fewer studies have explored the global effects of chronic stress on the brain’s structural and functional integrity in depressed patients. Cortical thickness is an important indicator associated with brain development (10). Given this, the present study aims to investigate the impact of chronic stress on cortical thickness in patients with depression, analyzing the characteristics of cortical thickness changes in these patients, thereby providing a basis for early diagnosis, condition assessment, and the development of personalized intervention strategies for clinical depression.

## 2 Materials and methods

This study was conducted in accordance with the principles outlined in the Declaration of Helsinki. The study protocol was reviewed and approved by the Institutional Ethics Committee of the First Hospital of Hebei Medical University. Written informed consent was obtained from all participants prior to their inclusion in the study.

### 2.1 Clinical data

From January 2023 to October 2024, 30 patients with depression and chronic stress (admitted to or treated at our hospital), 30 patients with depression without chronic stress (admitted to or treated at our hospital), and 30 healthy controls undergoing health check-ups at our hospital during the same period and matched by age and sex were recruited. Participants were assigned to the Chronic Stress Depression Group, the Depression Group, or the Control Group, respectively.

**Inclusion criteria for depression:**

1. Diagnosis of major depressive episode according to the Diagnostic and Statistical Manual of Mental Disorders, Fifth Edition (DSM-V) (11).
2. Hamilton Depression Rating Scale-17 (HAMD-17) (12) score ≥ 18.
3. Wechsler Intelligence Scale (13) score > 90.
4. Age between 20 and 60 years.
5. Patients receiving initial treatment for depression (treatment-naive).

**Exclusion criteria:**

1. Participants currently receiving psychotherapy or other pharmacological treatments.
2. Organic brain lesions.
3. Neurological diseases or severe systemic diseases.
4. Co-morbidities including schizophrenia, substance use disorders, intellectual disabilities, and other Axis I and II disorders in DSM-V.
5. History of alcohol or drug abuse.
6. Patients who had difficulty communicating or cooperating; presence of cardiac pacemakers or metal implants.
7. Pregnant or lactating women.
8. Malignant tumors or hematologic diseases.
9. Incomplete clinical data.
10. Participation in other clinical trials.

The study protocol complies with the ethical principles outlined in the Declaration of Helsinki by the World Medical Association.

### 2.2 Treatment methods

The prognosis and follow-up of patients in both the Chronic Stress Depression Group and the Depression Group were evaluated in a blinded manner by two or more attending physicians, who determined the treatment plan based on the individual patient’s condition. Both groups received oral Fluvoxamine Maleate (Zhuhai Free Trade Zone Lizhu Synthetic Pharmaceutical Co., Ltd., batch number: 20160209) at an initial dose of 50 mg/day, along with psychological intervention. The psychological intervention, primarily consisting of supportive psychotherapy, was provided one-on-one, conducted three times a week, 40 minutes per session, for a continuous 2-month period. Fluvoxamine Maleate is commonly used in China for treating depression and obsessive-compulsive disorder (OCD) and is considered a first-line treatment for depression. Patients included in this study did not present with comorbid OCD symptoms. Assessments were conducted immediately following this 2-month treatment period.

### 2.3 Observation indicators

#### 2.3.1 Chronic stress status

The chronic stress status of patients was assessed using the Life Events Scale (LES) (14) 1 day before treatment and 2 months after treatment initiation. The LES includes three areas: family life (28 items, e.g., marital conflict, illness of a close relative), work/study (13 items, e.g., job loss, major exam failure), and social life (7 items, e.g., conflict with neighbours, loss of a close friend), totaling 48 common life events. The total score for life event stress is calculated as the sum of positive and negative event stimulation scores. Each of the 48 events was rated by the subject based on personal experience over the past year, considering event nature, impact, and duration. The impact score for each event ranges from 0 to 4, and the duration score ranges from 1 to 4. The stimulation score for a single event is calculated as: impact score × duration score × event frequency. The total score is the sum of positive and negative event stimulation scores. A higher score indicates stronger chronic stress, and a score greater than 100 indicates the presence of chronic stress. While the LES provides a quantitative measure of life event stress, the threshold of 100 was adopted based on common practice in Chinese regional studies utilizing the LES developed by Yang and Zhang (1993), which emphasizes culturally relevant stressors and operationalizes chronic stress through cumulative severity scores (15, 16). However, this threshold lacks universal validation and should be interpreted within the context of its regional application; it serves as an operational definition for participant grouping in this study.

#### 2.3.2 Hamilton depression rating scale

The severity of depression symptoms was assessed using the Hamilton Depression Rating Scale-17 (HAMD-17) (10) 1 day before treatment and 2 months after treatment initiation. Developed by Hamilton in 1960, the HAMD-17 evaluates the severity of depressive symptoms across 17 items. Each item is rated from 0 to 4, with higher scores indicating more severe depression. This scale is widely used in clinical practice, and its total Cronbach’s alpha coefficient is 0.714.

#### 2.3.3 Neuroimaging examination

Neuroimaging was conducted 1 day before treatment and 2 months after treatment initiation. (1) Image Acquisition: MRI scans were performed using a Philips 3.0T MR imaging system (Philips Medical Systems NV, The Netherlands) with an 8-channel coil. Structural MRI images were obtained using a 3D spoiled gradient echo (SPGR) sequence with the following scanning parameters: TR 8.5 ms, TE 3.4 ms, flip angle 12°, slice thickness 1 mm, single excitation, field of view 24 cm × 24 cm, matrix 256 × 256, voxel size 0.47 mm × 0.47 mm × 1 mm, and a total of 156 axial images of the whole brain. (2) Image Processing: Cortical surface construction was performed using FreeSurfer v5.3.0 software. The images were segmented into gray matter, white matter, and cerebrospinal fluid. The gray-white matter boundary and pial surface were identified, and manual corrections were made to adjust for minor topological inaccuracies. After local corrections and smoothing of the edges, the initial cortical surface images were obtained. Deformation algorithms were used to reconstruct the pial surface. Cortical thickness was calculated at each vertex as the shortest distance from the gray-white matter boundary to the pial surface. Individual subject surfaces were registered to a common spherical space, and cortical thickness maps were smoothed using a Gaussian kernel of 15 mm full-width at half-maximum (FWHM). Parcellation into distinct anatomical regions was performed using the Desikan-Killiany (DK) atlas for subsequent region-of-interest (ROI) analysis.

#### 2.3.4 Follow-up

All patients were intended to be followed for up to 12 months post-discharge. Clinical remission was defined as a continuous 3-month period with a HAMD-17 score of less than 17, at which point follow-up could be terminated. For patients who did not meet this criterion, follow-up continued until 12 months after discharge. The time of depressive relapse was also recorded.

### 2.4 Quality control

A total of 36 patients with depression and chronic stress were initially recruited, of which 6 patients were lost to follow-up (1 due to natural death and 5 due to refusal to continue follow-up as they moved to other locations). For patients with depression without chronic stress, 38 were initially recruited, and 8 were lost to follow-up (all 8 refused to continue follow-up as they moved to other locations). A final total of 30 patients with chronic stress and depression and 30 patients with depression without chronic stress were included in the analysis. All participants completed questionnaires and scales at our hospital under the guidance of research team members, who provided standardized instructions and resolved any queries. Team members were also responsible for collecting and reviewing the questionnaires and scales. DSM-V diagnosis, HAMD-17 scores, and Wechsler Intelligence Scale scores were assessed for each participant by two trained attending physicians.

### 2.5 Statistical methods

Data were processed using SPSS 21.0 statistical software. Normally distributed continuous variables were expressed as mean ± standard deviation (mean ± SD), and group comparisons were made using independent samples t-tests or one-way analysis of variance (ANOVA), followed by Tukey’s HSD *post-hoc* tests where appropriate for pairwise comparisons. Categorical variables were expressed as frequencies (percentages) [n (%)] and compared using the chi-square test or Fisher’s exact probability test. Multiple comparisons for the cortical thickness analyses across different brain regions were corrected using the False Discovery Rate (FDR) method. The comparison of mean time to relapse between the two patient groups specifically utilized an independent samples t-test. A p-value of <0.05 was considered statistically significant.

## 3 Results

### 3.1 Comparison of general characteristics among the three groups

There were no significant differences in age, sex, BMI, disease duration, smoking history, alcohol consumption history, educational level, or employment status between the Chronic Stress Depression Group, Depression Group, and Control Group (P > 0.05), as shown in **Table 1**.

**Table 1:** Comparison of general characteristics among the three groups.

Item	Chronic stress depression group (n=30)	Depression group (n=30)	Control group (n=30)	t/F/χ^2^	P
Gender (Male) (n)	9	8	10	0.317	0.853
Age (years)	39.09 ± 9.81	38.87 ± 9.64	41.24 ± 9.95	-0.088	0.931
BMI (kg/m²)	22.39 ± 3.21	22.65 ± 3.42	22.16 ± 3.39	0.304	0.763
Disease Duration (months)	23.19 ± 4.23	23.08 ± 4.76	–	-0.095	0.925
Smoking History (n)	4	6	5	0.480	0.787
Alcohol History (n)	5	3	2	1.575	0.455
Education Level (n)				1.378	0.848
- College and Above	7	9	6		
- High School/Technical School	14	15	16		
- Junior High School or Below	9	6	8		
Employment Status (n)				1.003	0.909
- Student	9	7	8		
- Unemployed	16	19	16		
- Employed	5	4	6		

### 3.2 Comparison of chronic stress status among the three groups

**Figure 1:**
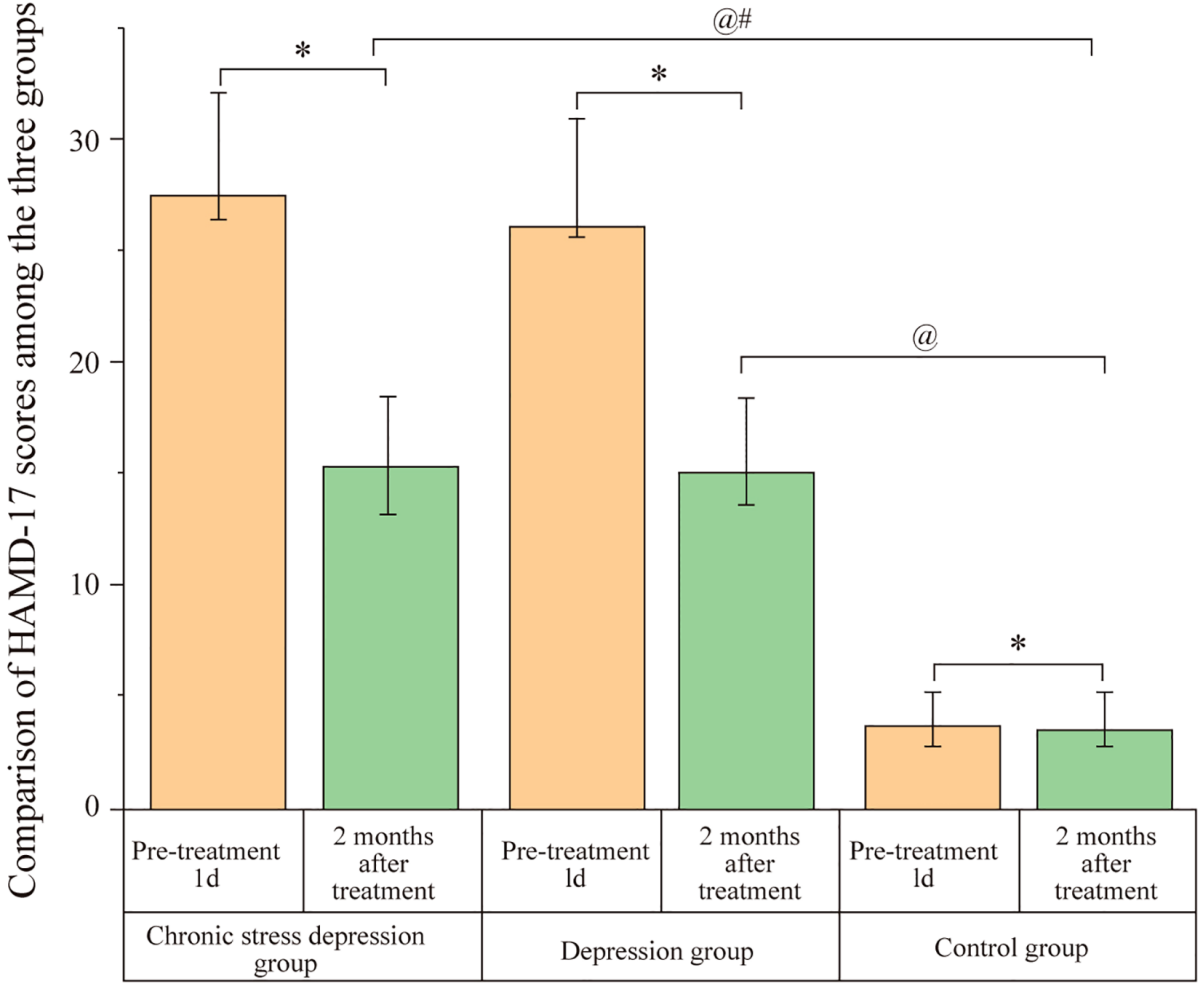
Comparison of chronic stress scores among the three groups. Scores represent the chronic stress status assessed by the Life Events Scale (LES) for the Chronic Stress Depression Group, Depression Group, and Control Group at baseline (1 day before treatment) and after 2 months of treatment. Data are likely presented as mean ± SD or similar. Annotations: ^@^P < 0.05 indicates a significant difference compared to the baseline score (1 day before treatment) within the same group; ^#^P < 0.05 indicates a significant difference compared to the Depression group at the same time point; ^*^P < 0.05 indicates a significant difference compared to the Control group at the same time point.

Before treatment (1 day), the Chronic Stress Depression Group exhibited significantly higher chronic stress scores compared to the Depression Group and the Control Group, with the Depression Group also showing higher scores than the Control Group. The differences were statistically significant (P < 0.05). After two months of treatment, both the Chronic Stress Depression Group and the Depression Group showed a reduction in their chronic stress scores. However, the Chronic Stress Depression Group continued to have higher chronic stress scores compared to the Depression Group and the Control Group, and the Depression Group also had higher scores than the Control Group. These differences remained significant (P < 0.05). The results are presented in **Figure 1**.

### 3.3 Comparison of HAMD-17 scores among the three groups

**Figure 2:**
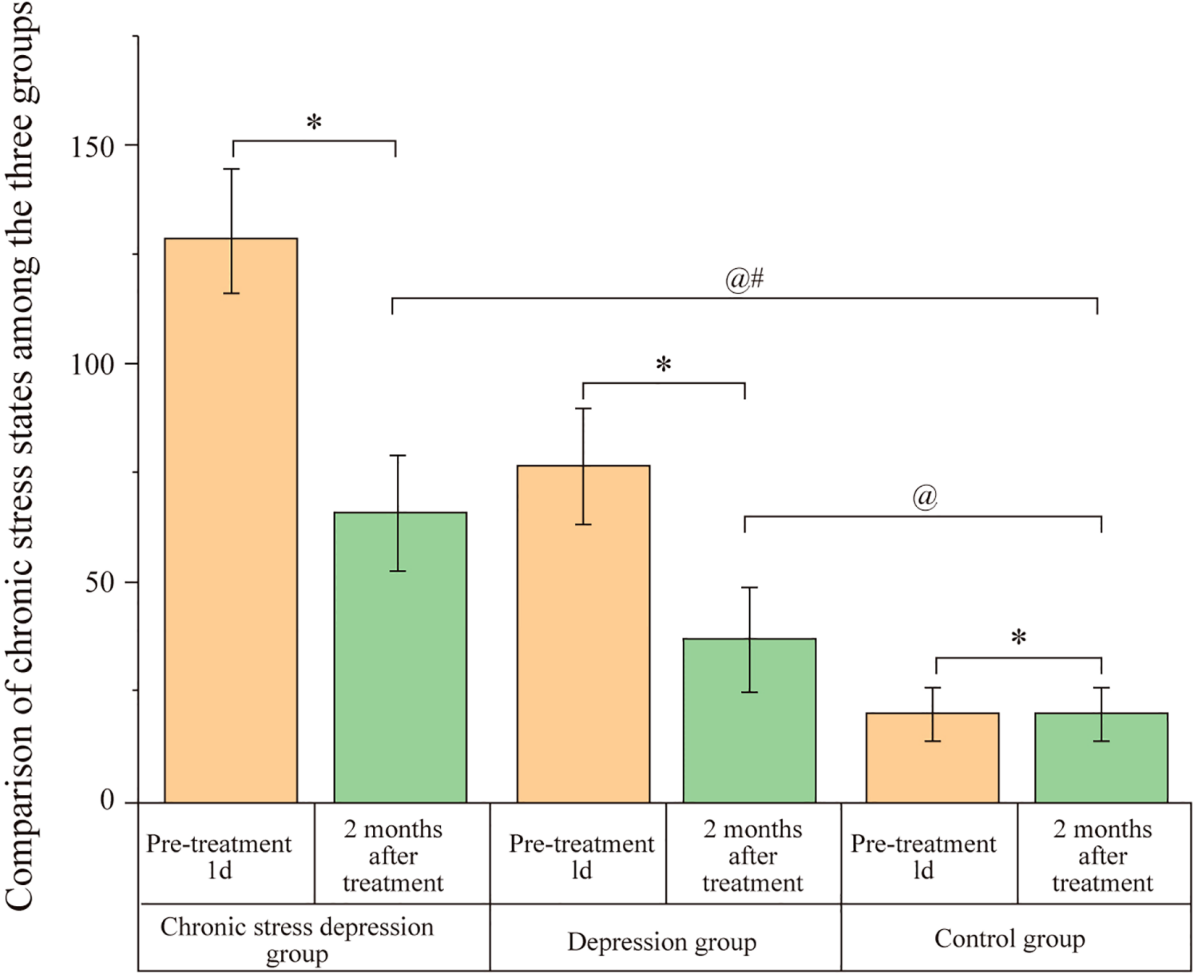
Comparison of HAMD-17 scores among the three groups. Scores represent the severity of depressive symptoms assessed by the Hamilton Depression Rating Scale-17 (HAMD-17) for the Chronic Stress Depression Group, Depression Group, and Control Group at baseline (1 day before treatment) and after 2 months of treatment. Data are likely presented as mean ± SD or similar. Annotations: ^@^P < 0.05 indicates a significant difference compared to the baseline score (1 day before treatment) within the same group; ^#^P < 0.05 indicates a significant difference compared to the Depression group at the same time point; ^*^P < 0.05 indicates a significant difference compared to the Control group at the same time point.

Before treatment (1 day), both the Chronic Stress Depression Group and the Depression Group had significantly higher HAMD-17 scores compared to the Control Group (P < 0.05). However, no significant difference was observed between the Chronic Stress Depression Group and the Depression Group (P > 0.05). After two months of treatment, both the Chronic Stress Depression Group and the Depression Group showed a significant decrease in HAMD-17 scores. Despite this, the HAMD-17 scores for both the Chronic Stress Depression Group and the Depression Group remained significantly higher than those of the Control Group (P < 0.05). There was no significant difference between the Chronic Stress Depression Group and the Depression Group (P > 0.05). The data are presented in **Figure 2**.

### 3.4 Comparison of cortical thickness among the three groups

**Figure 3:**
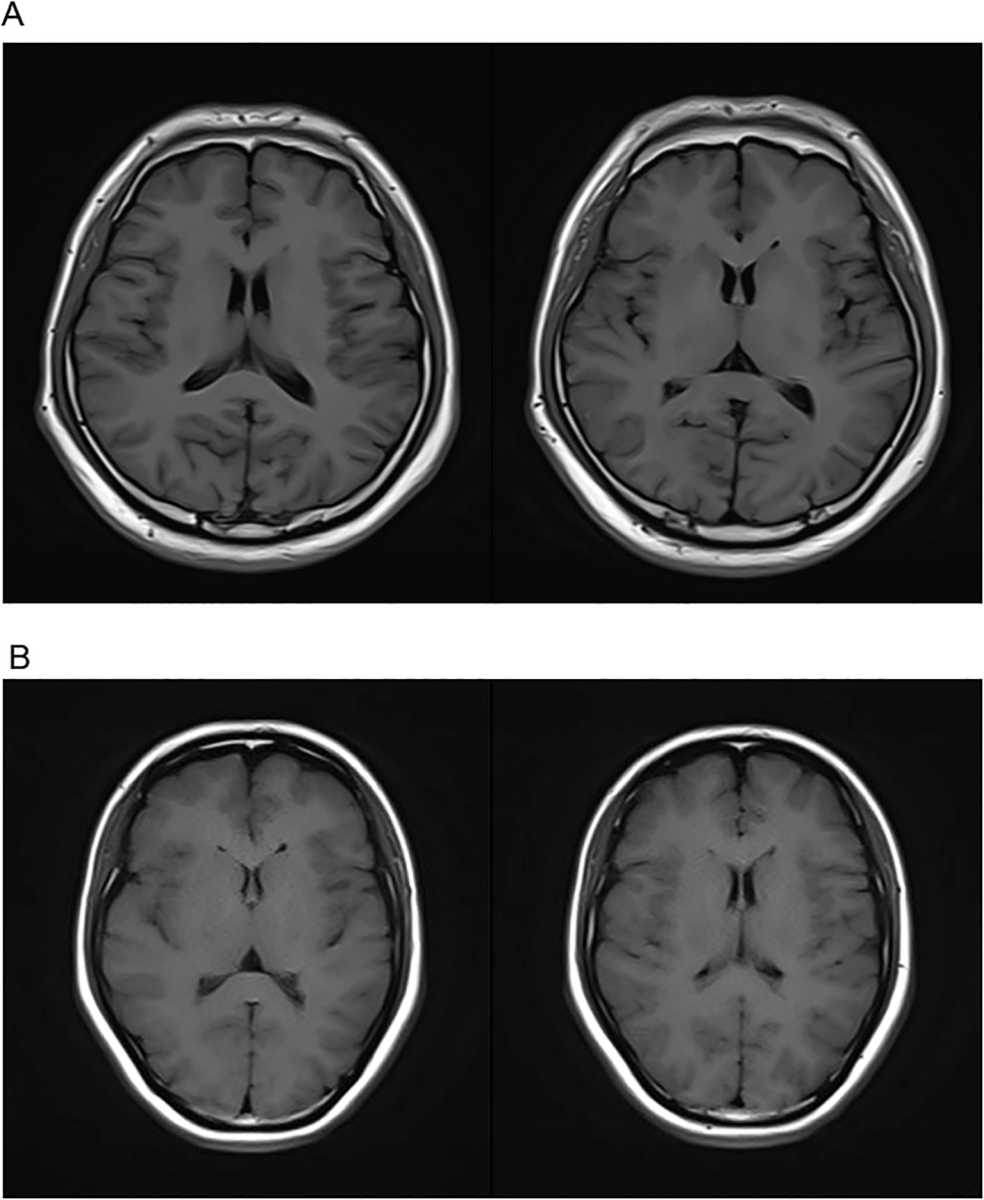
Representative axial T1-weighted MRI images. **(A)** Images from a healthy control (HC) participant. **(B)** Images from a patient with depression (pre-treatment), illustrating general brain structure.

Before treatment (1 day), the Chronic Stress Depression Group and the Depression Group showed significantly lower cortical thickness in the left prefrontal triangular area, frontal gyrus, precuneus, middle temporal gyrus, and cingulate gyrus, as well as in the right superior frontal gyrus, precuneus, and inferior frontal gyrus compared to the Control Group. Furthermore, the average cortical thickness in the Chronic Stress Depression Group was lower than in the Depression Group in the aforementioned brain regions, with significant differences (P < 0.05). Representative MRI scans illustrating the general brain structure from a healthy control and a patient with depression are shown in **Figure 3**, providing a qualitative context for the quantitative neuroimaging analyses performed. Two months after treatment, both the Chronic Stress Depression Group and the Depression Group showed increased cortical thickness in the same regions. However, despite this increase, the cortical thickness in both groups remained lower than in the Control Group (P < 0.05). No significant differences were found between the Chronic Stress Depression Group and the Depression Group in terms of cortical thickness (P > 0.05). The detailed comparison of cortical thickness for specific regions at both time points is shown in **Table 2**.

**Table 2:** Comparison of cortical thickness among the three groups (Mean ± SD, mm).

Region	Time	Chronic stress depression group (n=30)	Depression group (n=30)	Control group (n=30)	F	P
Left Hemisphere						
Prefrontal Triangular Area	Pre-treatment (1 day)	2.03 ± 0.19#*	2.39 ± 0.21*	2.65 ± 0.22	6.963	<0.001
	Post-treatment (2 months)	2.43 ± 0.16#*	2.51 ± 0.14*	2.62 ± 0.17	2.061	0.044
Frontal Gyrus	Pre-treatment (1 day)	1.92 ± 0.13#*	2.27 ± 0.12*	2.58 ± 0.16	10.836	<0.001
	Post-treatment (2 months)	2.35 ± 0.14#*	2.43 ± 0.15*	2.59 ± 0.14	2.136	<0.001
Precuneus	Pre-treatment (1 day)	2.08 ± 0.21#*	2.36 ± 0.24*	2.69 ± 0.22	4.809	<0.001
	Post-treatment (2 months)	2.43 ± 0.13#*	2.51 ± 0.11*	2.70 ± 0.13	2.573	0.013
Middle Temporal Gyrus	Pre-treatment (1 day)	2.32 ± 0.24#*	2.58 ± 0.27*	2.80 ± 0.26	3.942	0.002
	Post-treatment (2 months)	2.64 ± 0.17#*	2.73 ± 0.15*	2.79 ± 0.17	2.174	0.034
Cingulate Gyrus	Pre-treatment (1 day)	1.97 ± 0.13#*	2.18 ± 0.17*	2.34 ± 0.19	5.375	<0.001
	Post-treatment (2 months)	2.20 ± 0.09#*	2.26 ± 0.13*	2.35 ± 0.10	2.078	0.042
Right Hemisphere						
Superior Frontal Gyrus	Pre-treatment (1 day)	2.43 ± 0.11#*	2.68 ± 0.13*	2.86 ± 0.20	8.041	<0.001
	Post-treatment (2 months)	2.73 ± 0.05#*	2.76 ± 0.06*	2.87 ± 0.03	2.104	0.040
Precuneus	Pre-treatment (1 day)	2.23 ± 0.15#*	2.48 ± 0.07*	2.67 ± 0.16	8.272	<0.001
	Post-treatment (2 months)	2.54 ± 0.05#*	2.58 ± 0.09*	2.68 ± 0.14	2.128	0.038
Inferior Frontal Gyrus	Pre-treatment (1 day)	1.78 ± 0.14#*	1.96 ± 0.12*	2.19 ± 0.13	5.347	<0.001
	Post-treatment (2 months)	2.08 ± 0.05#*	2.12 ± 0.07*	2.20 ± 0.14	2.547	<0.001
Average Cortical Thickness	Pre-treatment (1 day)	1.95 ± 0.12#*	2.29 ± 0.09*	2.48 ± 0.13	13.405	<0.001
	Post-treatment (2 months)	2.34 ± 0.04#*	2.39 ± 0.06*	2.49 ± 0.05	3.798	<0.001

Compared to the Depression Group (#P < 0.05), compared to the Control Group (*P < 0.05).

### 3.5 Follow-up results

All patients were followed up for 12–14 months, with a mean follow-up duration of (12.86 ± 1.64) months. At the final follow-up, 28 patients in the Chronic Stress Depression Group had a HAMD-17 score < 17, and 29 patients in the Depression Group had a HAMD-17 score < 17. There was no significant difference in treatment outcome (based on HAMD-17 score < 17 at final follow-up) between the two groups (χ² = 0.351, P = 0.554). However, the time to depressive relapse was significantly shorter in the Chronic Stress Depression Group (5.39 ± 2.31 months) compared to the Depression Group (8.28 ± 2.51 months) (t = 4.656, P < 0.001).

## 4 Discussion

Depression is an emotional disorder primarily characterized by significant and persistent mood lows, loss of pleasure, lack of energy, and a series of other symptoms. It is associated with high morbidity, high suicide rates, high relapse rates, and high disability rates. The main treatment methods for depression include pharmacotherapy, psychotherapy, and physical therapy. Currently, pharmacotherapy remains the primary clinical treatment for depression, with fluvoxamine being one of the commonly used medications. Fluvoxamine belongs to the SSRI (Selective Serotonin Reuptake Inhibitor) class of antidepressants. It strongly inhibits the reuptake of serotonin (5-HT) in the central nervous system, effectively increasing the concentration of serotonin in the synaptic cleft, which contributes to shortening recovery time and enhancing its therapeutic effect (17). Additionally, psychotherapy is often combined, which has been shown to improve treatment outcomes. Goryunov et al. (18) suggested that fluvoxamine effectively treats depression. Dardas et al. (19) indicated that fluvoxamine could reduce inflammatory markers in patients with depression, thus contributing to its therapeutic effect. Yilmaz et al. (20) pointed out that psychological interventions could be effective in treating depression. Therefore, this study uses fluvoxamine combined with psychological interventions for the treatment of depression.

Stress refers to the adaptive response of the body to external stimuli. While the body can often gradually adapt, chronic stress can damage the central nervous system, affecting its adaptive capacity, potentially causing various physiological and psychological issues. Previous studies have found (8) that individuals with major depressive disorder typically experience more stressful life events before the onset of depression, suggesting that chronic stress may play an important role in the occurrence and development of depression. Banasr et al. (21) noted that depression is a stress-related disorder, with cortical-limbic changes being associated with depressive symptoms. Xie et al. (22) suggested that Negative Life Events (NLEs) and alexithymia could serve as predictive factors for depressive symptoms. The results of this study show that, at baseline (1 day before treatment), the Chronic Stress Depression Group had significantly higher chronic stress scores than both the Depression Group and the Control Group, and the Depression Group also had significantly higher scores than the Control Group (P < 0.05), indicating that chronic stress exposure was more prominent in the chronic stress depression patients.

Regarding the relationship between chronic stress and depression, previous studies have presented different viewpoints. Some suggest that stress is the direct cause of depression (direct cause model); others propose that stress is merely one of the triggers, which may activate an underlying predisposition (vulnerability-stress model) (23–25); still others argue that chronic stress and depression influence each other (mutual influence model) (26). After two months of treatment, the Chronic Stress Depression Group and the Depression Group both showed a reduction in chronic stress scores. However, the Chronic Stress Depression Group still had significantly higher scores than the Depression Group and Control Group, and the Depression Group had significantly higher scores than the Control Group (P < 0.05). This suggests that fluvoxamine combined with psychological intervention can reduce chronic stress scores and alleviate the chronic stress state in both groups of depressed patients. However, the persistently higher scores in the Chronic Stress Depression group, even after treatment, might be explained by several factors: while fluvoxamine modulates neurotransmitters and psychological intervention improves cognitive patterns and reduces subjective stress perception, long-term stress may induce more persistent structural brain changes (e.g., heightened amygdala sensitivity, reduced prefrontal regulatory function, hippocampal volume reduction) requiring longer neural repair times. Furthermore, patients might remain exposed to chronic environmental stressors; the combined therapy may enhance coping abilities rather than eliminating the stressor itself. Although both groups showed reductions post-treatment, the persistently higher LES scores in the Chronic Stress Depression group, significantly differing from the Depression-only group, likely reflect not just a statistical difference but a clinically meaningful higher burden of ongoing or recent life stressors. This suggests that even with symptom improvement, the underlying environmental or perceived stress context remains distinct and potentially more challenging for these individuals, contributing to the observed differences in relapse rates.

Zhang et al. (27) noted that effective treatment could reduce depression-related scores, such as HAMD, and improve depressive symptoms. Other studies, such as those by Li et al. (28) and Shi et al. (29), have highlighted the efficacy of fluvoxamine in reducing HAMD-17 scores during the acute phase of depression and potentially being particularly effective for depression with anxious distress features. In this study, after two months of treatment, both the Chronic Stress Depression Group and the Depression Group showed a significant decrease in HAMD-17 scores, but their scores remained significantly higher than those of the Control Group (P < 0.05). No significant difference in HAMD-17 scores was observed between the Chronic Stress Depression Group and the Depression Group post-treatment (P > 0.05). This indicates that fluvoxamine combined with psychological intervention effectively alleviates depressive symptoms in both chronic stress depression and standard depression patients, consistent with previous findings (30). These results support a comprehensive treatment model combining medication to manage core symptoms with psychological intervention to address stress-related triggers, offering practical evidence for optimizing clinical management of depression.

Previous studies have indicated that the dorsolateral prefrontal cortex (DLPFC) and the cingulate gyrus are involved in the monitoring and reporting of emotions, language, and mental states (31, 32). The anterior cingulate gyrus (ACC) also participates in the regulation of cognition and emotions, as well as in the perception of the external world and the monitoring of one’s own behavior. The posterior cingulate gyrus (PCC) is responsible for emotional processing, and thus is associated with cognitive processing and emotional regulation in patients with depression. The precuneus is involved in integrating information obtained from the external environment with emotions, and in extracting situational memories; therefore, cortical thinning in these regions may be linked to abnormal emotional integration in depression patients.

Other studies have shown that antidepressants can inhibit microglial activation, thereby controlling the immune response in the central nervous system (33). This regulatory effect is directly and indirectly related to the efficacy of antidepressant treatment. Chronic stress can promote microglial activation and enhance the interaction between neurons and glial cells. Moreover, research has found that microglial activation in the anterior cingulate gyrus is increased in patients with depression, and microglial activation in the frontal white matter is significantly higher in individuals with depression who died by suicide (34). Based on the above analysis, it can be hypothesized that chronic stress may play a role in the onset, exacerbation, and treatment efficacy of depression via neuroinflammatory pathways.

Additionally, some studies have suggested that cortical thickness can reflect neural fiber density, making it an important indicator of brain developmental changes (35). Chen et al. (36) proposed that effective treatment can improve cortical structure in patients with depression. Further research, such as by Yang et al. (37) and Wang et al. (38), suggests that effective interventions can alter brain region volumes and connectivity, and dynamically modulate brain activity (e.g., enhancing middle frontal gyrus activity while reducing middle occipital gyrus activity), providing a neuroimaging basis for symptom improvement. The results of this study show that before treatment, both patient groups exhibited reduced cortical thickness in several regions compared to controls, with the chronic stress group showing significantly greater reductions than the non-stress group. After two months of treatment, both the Chronic Stress Depression Group and the Depression Group exhibited increased cortical thickness in the left pars triangularis, pars opercularis (frontal regions), the precuneus, the middle temporal gyrus, and the cingulate gyrus, as well as the right superior frontal gyrus, the precuneus, and the inferior frontal gyrus. However, the cortical thickness in these regions remained significantly lower in both patient groups compared to the Control Group (P < 0.05). There was no significant difference between the Chronic Stress Depression Group and the Depression Group post-treatment (P > 0.05).

These findings suggest that patients with depression exhibit cortical thinning (or abnormal changes) in multiple brain regions, and that chronic stress exacerbates these alterations. These cortical abnormalities, potentially reflecting underlying neural structural remodeling (such as reduced neuronal density, synaptic loss, or glial dysfunction), may constitute an important pathological basis for depression. Given that the affected regions are involved in emotional regulation, cognitive processing, and situational memory integration, structural thinning likely contributes to symptoms like emotional dysregulation, cognitive deficits (e.g., attention, memory issues), and negative affect, consistent with previous studies (36). Furthermore, the results indicate that fluvoxamine combined with psychological intervention can ameliorate cortical thinning in patients with depression, including those exposed to chronic stress.

The follow-up results of this study also show that the time to depressive relapse in the Chronic Stress Depression Group was significantly shorter than in the Depression Group (P < 0.001). This suggests that patients with chronic stress and depression experience faster recurrence of depressive episodes and have a worse prognosis than those with depression alone. While this study highlights the role of chronic stress, it is acknowledged that other factors, such as the degree of initial treatment response or residual symptom burden, could also influence relapse times. The mechanisms by which chronic stress accelerates relapse may involve persistent HPA axis dysregulation, heightened neuroinflammatory sensitivity, or impaired neural plasticity affecting long-term mood regulation, warranting further investigation. While effective interventions can improve brain structure and restore neural plasticity, thereby improving symptoms, the observation that cortical thickness did not fully normalize to healthy control levels post-treatment may partially explain the heightened relapse risk, particularly in the chronic stress group.

However, this study has limitations. The sample size is relatively small, and the follow-up duration is short. Additionally, data on cortisol levels, a key biological marker of stress, were not collected. Furthermore, the analysis of relapse relied on comparing mean times rather than employing survival analysis techniques like Kaplan-Meier, which could provide a more nuanced view of relapse patterns. These factors may lead to potential bias in the results. Future studies should include larger sample sizes, longer follow-up periods, incorporate biological stress markers and appropriate survival analysis methods, and potentially collaborate with other hospitals to further confirm the findings of this study.

In summary, fluvoxamine combined with psychological intervention can reduce chronic stress scores in both chronic stress depression patients and depression patients, alleviate the chronic stress state, improve depressive symptoms, and ameliorate cortical thinning in multiple brain regions. Furthermore, chronic stress appears to exacerbate cortical thinning in various brain regions in depression patients, likely participating in the pathogenesis and progression of depression through multiple mechanisms, which is closely related to patient prognosis.

## Data Availability

The raw data supporting the conclusions of this article will be made available by the authors, without undue reservation.
